# A Modified Anteromedial Approach for Exposure of Coronoid Fractures: An Anatomical Cadaver Study

**DOI:** 10.1155/2019/6872948

**Published:** 2019-03-04

**Authors:** Fei Yang, Kai Feng, Yuehua Sun, Kerong Dai, Xiaoqing Wang, Jian Tang

**Affiliations:** Shanghai Key Laboratory of Orthopaedic Implant, Department of Orthopaedics, Shanghai Ninth People's Hospital, Shanghai Jiao Tong University School of Medicine, 639 Zhizaoju Road, Shanghai 200011, China

## Abstract

The previous anterior approach to the elbow provides a limited exposure of the coronoid process. The aim of this study was to identify the optimal exposure interval for coronoid fractures from the anterior aspect of elbow. We exposed the coronoid process on twelve fresh-frozen cadaveric elbows from the anteromedial aspect of the elbow. The dissection intervals used were the novel brachial artery-median nerve interval (B-M interval) and the previously reported biceps tendon-brachial artery interval (B-B interval). Access to key anatomic landmarks around the coronoid process was assessed and the exposed surface area of coronoid process was calculated in each specimen. The average exposed surface area of coronoid process was 2.26 times greater with the B-M interval (4.58 cm^2^) compared with the B-B interval (2.03 cm^2^) (p < 0.05). All key anatomic landmarks around the coronoid process were directly visualised via the B-M interval in each specimen. In conclusion, the anteromedial approach through the B-M interval provides a more extensive exposure of the coronoid process than the traditional B-B interval. This new approach can be useful for the fixation of coronoid fractures.

## 1. Introduction

Secure fixation of the coronoid fragment is a critical element in the treatment of elbow injuries. However, the usual surgical approaches to the elbow are not optimal to expose and fix coronoid fractures. The medial approach uses three intervals in the flexor-pronator musculature to gain access to coronoid process and requires an extensive dissection and increases the risk of residual instability, heterotopic ossification, elbow stiffness, and ulnar nerve palsy [1–3]. Additionally, it is usually difficult to fix the fragments of the coronoid process from the medial incision, because of the often difficult elevation of the thick flexor-pronator mass [4]. The lateral exposure readily provides access to the radial head and lateral collateral ligament (LCL), but not the coronoid process. Three types of coronoid fracture were originally described by Regan and Morrey [5], and the exposure approach of coronoid fracture depends on the size of the fragment, the anatomic location, and the presence of elbow instability. Visualisation of the coronoid fracture is easy when the radial head is comminuted and requires removal [6, 7]. However, when only a portion of the radial head is fractured, it is quite difficult to expose and fix the coronoid fracture through a lateral approach. The posterior approach with subcutaneous lateral and medial extensions can be used to treat large coronoid fragments associated with LCL rupture or transolecranon fracture-dislocations [3, 8, 9]. For the isolated coronoid fracture, the posterior approach is not generally recommended because of the degree of soft tissue dissection required.

Generally, the anterior approach is avoided for coronoid fractures because it may result in the restriction of elbow extension and the risk of iatrogenic injury to the neurovascular structures in the anterior of elbow. However, several surgeons reported clinical cases related to the anterior approach to coronoid fractures [10–13]. These studies showed some obvious advantages of the anterior approach: (1) providing excellent visualisation of and most direct access to the coronoid fracture; (2) allowing anatomic reduction of the fracture and easier placement of screws; (3) avoiding a large amount of soft tissue dissection; (4) avoiding damaging the flexor-pronator muscle mass and the ulnar nerve.

In these studies, the dissection intervals used were between the biceps tendon and the brachial artery (B-B interval). However, because of the anatomical characteristics of the neurovascular structures in the anterior of elbow, the exposure of coronoid fractures was limited considerably. In their paper, Zhu et al. pointed out that the radial recurrent artery restricted the exposure of coronoid fragment when through the B-B interval [4]. They believed that when using the anterior approach, there were some difficulties to optimally handle the artery branches to harvest more operating space. Because of the limitations of the B-B interval, we proposed the brachial artery-median nerve interval (B-M interval) for the exposure of coronoid process in this study.

**Figure 1:**
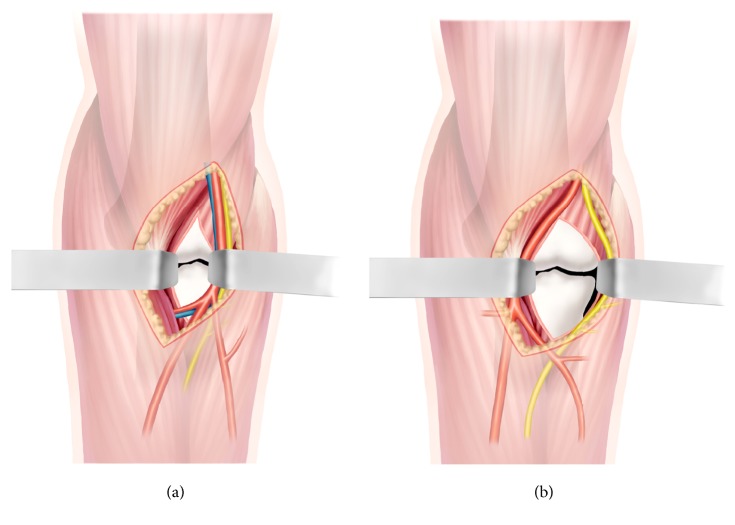
(a) The B-B interval: the anteromedial and basal structures of the coronoid process could not be completely exposed through the B-B interval without cutting the radial recurrent artery. (b) The B-M interval: all key anatomic landmarks were directly visualised with the B-M interval.

The goal of this research was to quantitatively assess the effect of exposure of the coronoid process, including key anatomic landmarks, using the B-M interval and traditional B-B interval (Figure 1). We hypothesized that the B-M interval could provide superior exposure of the coronoid process without cutting artery branches in the anteromedial elbow. To our knowledge, such quantification and description comparing these two surgical approaches to the coronoid process have not been previously reported in the literature.

## 2. Materials and Methods

### 2.1. Surgical Procedure

This study was approved by the local Ethics Committee. Twelve thawed, fresh-frozen cadaveric upper-extremity specimens were used in this study. None of these specimens had evidence of previous surgery, trauma, or osteoarthritis to the elbow. All dissections were performed by a single board-certified orthopedic trauma surgeon.

**Figure 2:**
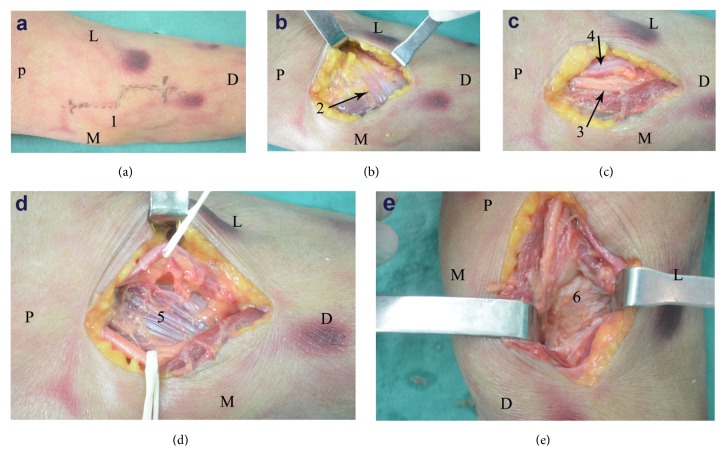
(a) A single curved incision (1) was made along the anteromedial aspect of the elbow. (b) The bicipital aponeurosis (arrow 2) was incised perpendicular to the aponeurotic fibers. (c) The neurovascular bundle in front of the elbow joint, which consists of median nerve (arrow 3) and brachial artery (arrow 4), was identified. (d) Brachialis muscle insertion (5) was exposed and split longitudinally between the retracted structures. (e) The coronoid process (6) was exposed through the split brachialis tendon. D, distal; L, lateral; M, medial; P, proximal.

The skin incision commenced 3 cm proximal to the cubital crease, along the medial border of biceps, and curved across the cubital crease, extending 3 cm distally along the midline of the forearm (Figure 2(a)). The bicipital aponeurosis was exposed underneath the subcutaneous tissue and incised perpendicular to the aponeurotic fibers (Figure 2(b)). The neurovascular bundle in front of the elbow joint, which consists of the median nerve and brachial artery, was identified (Figure 2(c)). In this study, the coronoid process was exposed through the brachial artery-median nerve interval (B-M interval) and biceps tendon-brachial artery interval (B-B interval) separately. Using a randomised crossover design for surgical sequence, the cadaveric limbs were then randomised to undergo either the B-M interval or the B-B interval surgical exposure first. Upon completion of the first approach, the limbs then underwent the latter approach, resulting in a total of 24 cadaveric dissections. The radial recurrent artery was not cut and ligated in any of the procedures.

When dissecting via the B-M interval, the brachial artery and concomitant veins were retracted laterally, and the median nerve was retracted medially. Brachialis muscle insertion was exposed between those retracted structures, and it was longitudinally split (Figure 2(d)). The area of coronoid process exposed was assessed through the split brachialis tendon (Figure 2(e)). During dissection via the B-B interval for the same limb, biceps tendon was retracted laterally, and the neurovascular bundle including brachial artery and median nerve was retracted medially. The visible area of coronoid process was assessed through the split brachialis tendon.

### 2.2. Data Collection

Upon completion of each exposure, visible access to specific anatomic landmarks was assessed. These included coronoid tip, anteromedial facet, coronoid base, and the sublime tubercle with its attachment of the anterior bundle of the medial collateral ligament (MCL).

Standard surgical retractors were used to demonstrate maximal exposure of the coronoid process. Digital pictures were taken perpendicular to the dissection and analyzed using a software program (AutoCAD 2010, Autodesk). A standardized measuring ruler was placed in the field of view to set the scale for the program. The scale was set using a standard distance of 1 cm. The exposed osseous area of the coronoid process was outlined using the software program, and the number of pixels contained within that area was calculated and converted to cm^2^ using the previously set scale. This method of measuring osseous surface area has been reported previously [14–16]. Statistical analysis was performed using a Wilcoxon signed rank sum test for nonparametric data, and significance was set at p < 0.05.

## 3. Results

The two described approaches were successfully performed in all specimens in sequential fashion. Specimen measurements after each approach are listed in Table 1.

The average surface area of coronoid process exposed was 4.58 cm^2^ (range 3.20–6.13, SD 0.99) using the B-M interval compared with 2.03 cm^2^ (range 1.09–3.92, SD 0.91) using the B-B interval (p < 0.05). The exposed area of the B-B interval to coronoid process is only about 44.3% of that of the B-M interval. It was noted that the exposure area of the coronoid process was considerably restricted by the radial recurrent artery, because it tethered the brachial artery and impeded it from being retracted medially when approaching through the B-B interval. The closer the radial recurrent artery was to the coronoid process, the less the brachial artery could be retracted medially, and the less the coronoid process was exposed.

All key anatomic landmarks were fully exposed with the B-M interval in each specimen. However, it was not effective to expose the anteromedial facet, coronoid base, and sublime tubercle through the B-B interval. Using the B-B interval, there was only complete visualisation of the anteromedial facet in five limbs (42%), the coronoid base in seven limbs (58%), and the sublime tubercle/anterior bundle of the MCL in three limbs (25%) (Table 2).

## 4. Discussion

Achievement of an adequate exposure, stable fixation, and minimisation of soft-tissue damage are required for successful surgical management of coronoid fractures. The purpose of this study was to report a novel approach for exposing the coronoid process. Major findings of the study include the following. (1) The neurovascular structures near the coronoid process have distinctive anatomical features, and based on these features we proposed a new B-M interval. (2) The skin incision and dissection of the B-M interval are different from the previous anterior approaches, and the exposed area is larger than the traditional B-B approach. (3) The B-M interval does not damage any branch of the anterior neurovascular bundle and avoids a large soft tissue dissection, which could expose all the important structures of coronoid process.

**Figure 3:**
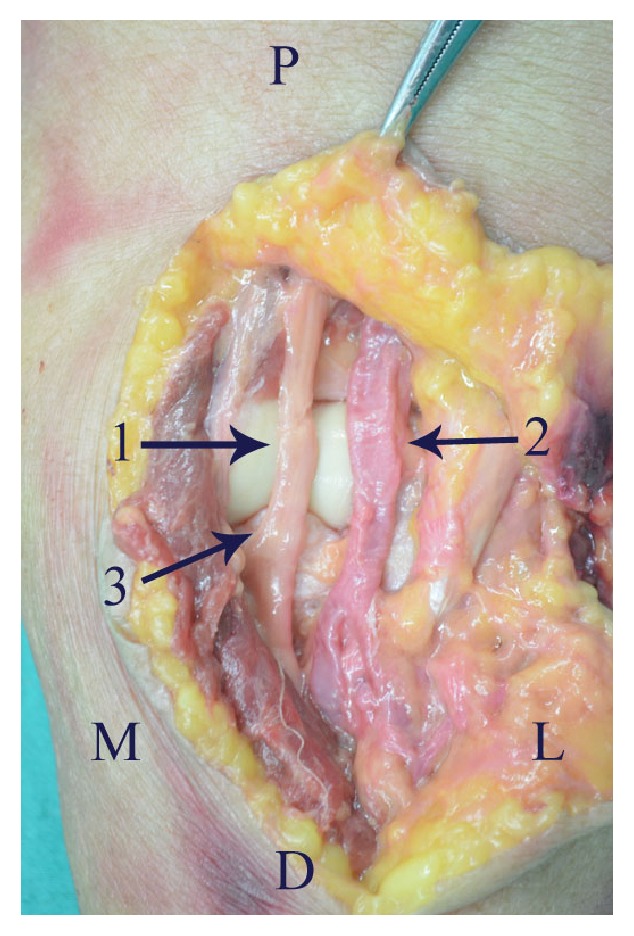
There was a natural interval between the median nerve (arrow 1) and brachial artery (arrow 2). Arrow 3 indicates the coronoid process. D, distal; L, lateral; M, medial; P, proximal.

**Figure 4:**
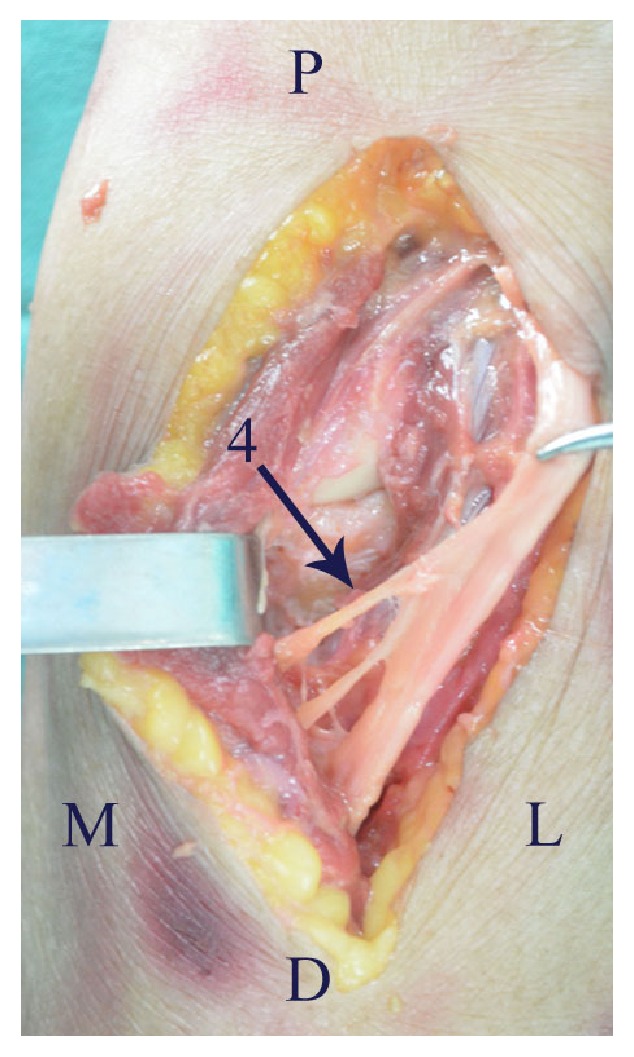
Some branches (arrow 4) went into the flexor-pronator muscle mass from the ulnar side of median nerve. D, distal; L, lateral; M, medial; P, proximal.

**Figure 5:**
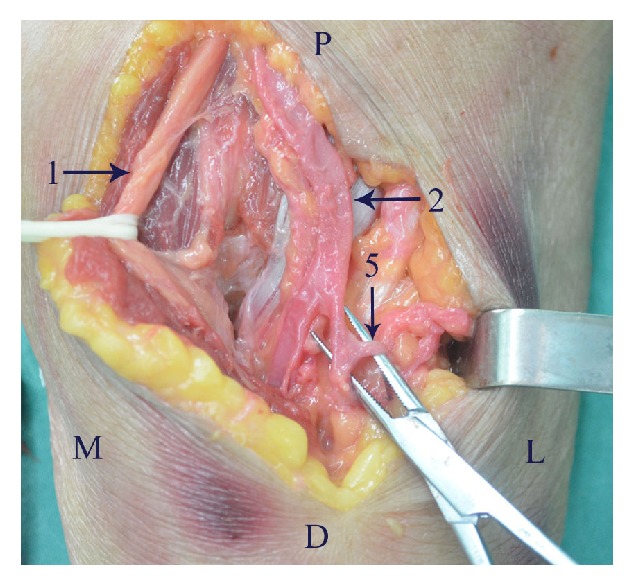
The radial recurrent artery (arrow 5) arose approximately 0.5cm from the origin of the radial artery and tethered and impeded brachial artery retraction medially. D, distal; L, lateral; M, medial; P, proximal.

The anterior approach provides excellent visualisation and the most direct access to a coronoid fracture. However, the anterior approach is avoided generally because of the risk of iatrogenic injury to the neurovascular structures in the anterior of elbow. During dissection of the neurovascular structures near coronoid process we found the following anatomical characteristics. There was a natural interval (B-M interval) between the brachial artery and median nerve (Figure 3). Above the cubital crease, the median nerve and brachial artery were superficial at the medial side of biceps, so the B-M interval was easy to identify. Below the cubital crease, the median nerve and brachial artery were covered by the flexor-pronator mass; thus it was difficult to identify the B-M interval. Therefore, it was suggested that the B-M interval is initially identified at the medial side of biceps, above the cubital crease, and then was dissected along the neurovascular bundle to the coronoid process. Some branches went into the flexor-pronator muscle mass from the ulnar side of median nerve (Figure 4). The radial recurrent artery arose approximately 0-0.5cm from the origin of the radial artery and restricted the retraction of the brachial artery medially (Figure 5). Notably, the closer the radial recurrent artery was to the coronoid process, the less the brachial artery could be retracted medially, and the less the coronoid process was exposed. The ulnar recurrent artery arose approximately 3-3.5cm from the origin of the ulnar artery, which was sufficiently far away from coronoid process to allow retraction of the brachial artery laterally.

In previous literature, Selesnick [10], Reichel [12], and Han [13] published case reports describing open reduction and internal fixation of the coronoid fracture through an anterior approach. The dissection interval used in each study was between the biceps tendon and the brachial artery (B-B interval). The biceps tendon was retracted laterally, and both brachial artery and median nerve were retracted medially. However, our detailed anatomic study showed that medial retraction of the brachial artery was restricted by the radial recurrent artery, which limited the exposure of coronoid process considerably. In the present study, we propose a novel dissection approach, via the B-M interval. Dissection via the B-M interval could protect the vascular tree in the anterior elbow. Furthermore, our data quantitatively confirmed that the B-M interval provides more extensive osseous exposure of the coronoid process than the B-B interval. Coronoid fractures may involve the following anatomic structures: the tip of the coronoid, anteromedial facet, coronoid base, and sublime tubercle with the attachment of the anterior bundle of the MCL. We found that the anteromedial and basal structures of the coronoid process could not be well exposed through the B-B interval without cutting the radial recurrent artery (Figure 1(a)). However, all key anatomic landmarks were directly visualised with the B-M interval in each specimen, indicating that this dissection interval is applicable to all types of coronoid fractures (Figure 1(b)).

This study had several limitations. First, the elasticity of cadaveric muscle was still different from that of normal muscle, which may not replicate the conditions in a true operation. Second, although we used advanced digital imaging software, it was limited by the fact that a two-dimensional image was attempting to represent a three-dimensional surface. Third, as pointed out in previous literature [14–16], the surgeon's view photograph may be a weakness, which may actually underestimate the surface area because manipulation of the retractors may provide even more osseous exposure.

In clinic, we performed the first operation using this anteromedial approach through the B-M interval in July 2013. From 2013 to 2018, 18 patients with coronoid fractures had been operated using this new approach in our hospital. These cases demonstrated that the plate, screws, and suture anchor could be easily placed to fix coronoid fractures through this approach. After operation, 16 patients were followed up and all of them achieved a full functional range of elbow motion for daily activities. We will report the treatment effect of these patients in another paper.

## 5. Conclusion

The anteromedial approach through the B-M interval provides 2.26 times more osseous exposure of the coronoid process than the B-B interval previously reported. The B-M interval could also provide adequate access to all key anatomic structures involving the coronoid process and we believe this approach can be considered for treating all types of coronoid fractures. Compared with other approaches, our approach avoids splitting the flexor-pronator mass and injuring the median nerve branches and the radial recurrent artery. So the anteromedial approach through the B-M interval is a good exposure for coronoid process visualization and possibly useful for the reduction and fixation of coronoid fractures.

## Figures and Tables

**Table 1:** Specimen measurements by dissection interval.

Cadaver No.	B-M Interval	B-B Interval
	Square Area Exposed (cm^2^)	Square Area Exposed (cm^2^)
1	4.103	1.656 (40.4%)
2	3.351	1.386 (41.4%)
3	5.565	2.052 (36.9%)
4	6.127	3.924 (64.0%)
5	5.014	2.711 (54.1%)
6	4.837	3.475 (71.8%)
7	5.723	2.237 (39.1%)
8	3.195	1.665 (52.1%)
9	3.286	1.595 (48.5%)
10	4.997	1.09 (21.8%)
11	4.76	1.439 (30.2%)
12	3.981	1.12 (28.1%)

The exposed area of the B-B interval to coronoid process is only about 44.3% (mean value) of that of the B-M interval.

**Table 2:** Anatomic landmarks identified with each interval.

Anatomic Landmarks	B-M Interval	B-B Interval
	n/12	n/12
Coronoid tip	12 (100%)	12 (100%)
Anteromedial facet	12 (100%)	5 (42%)
Coronoid base	12 (100%)	7 (58%)
Sublime tubercle	12 (100%)	3 (25%)

## Data Availability

The data used to support the findings of this study are available from the corresponding author upon request.
